# London Lancet

**Published:** 1863

**Authors:** 


LONDON LANCET.—This excellent monthly comes, as usual, richly laden with matter of practical interest fresh from our English brethren. Re-printed by JAMES HEALD, 113 Nassau Street, New York.

